# Living with myocardial ischaemia and no obstructive coronary arteries: a qualitative study

**DOI:** 10.1136/openhrt-2023-002569

**Published:** 2024-02-08

**Authors:** Helen Humphreys, Danielle Paddock, Sarah Brown, Colin Berry, Aynsley Cowie, Susan Dawkes, Simon Nichols

**Affiliations:** 1 Centre for Behavioural Science and Applied Psychology, Sheffield Hallam University, Sheffield, UK; 2 Cardiovascular Care Partnership, London, UK; 3 International Heart Spasms Alliance, London, UK; 4 BHF Glasgow Cardiovascular Research Centre, University of Glasgow, Glasgow, UK; 5 Cardiac Rehabilitation, NHS Ayrshire and Arran, Kilmarnock, UK; 6 School of Nursing, Midwifery and Paramedic Practice, Robert Gordon University, Aberdeen, UK; 7 School of Nursing and Midwifery, Edith Cowan University, Joondalup, Western Australia, Australia

**Keywords:** Microvascular Angina, Health Services, Coronary Artery Disease

## Abstract

**Objective:**

To explore the lived experience of people with myocardial ischaemia with no obstructive arteries.

**Design:**

Qualitative study using semistructured interviews.

**Setting:**

Telephone interviews with 17 participants living in the UK.

**Participants:**

17 people (2 males, 15 females; aged 31–69 years) with a presumed or confirmed diagnosis of myocardial ischaemia with no obstructive arteries, recruited via social media and online patient-led support forums.

**Results:**

Five themes were generated. Theme 1 describes the wide range of experiences that participants described, particularly the frequency and intensity of symptoms, and the uncertainty and fear that symptoms commonly provoked. Theme 2 describes the major impact on social relationships, employment and other aspects of everyday life. Theme 3 illustrates challenging and traumatising experiences participants described around pathways to diagnosis and accessing medical support. Theme 4 highlights the lack of consensus and clarity that participants had been confronted with around treatment and management. Theme 5 describes coping and supportive strategies valued by participants.

**Conclusions:**

This study provides insight into the challenges of living with myocardial ischaemia with no obstructive arteries. Findings highlight the significant psychological impact on people living with these conditions and the need for improvements in diagnosis, support and long-term management.

**WHAT IS ALREADY KNOWN ON THIS TOPIC**

- There is an increasing awareness of the prevalence of ischaemia with non-obstructive coronary arteries (INOCA) and ongoing work to improve its diagnosis. However, very little is known about the lived experience of people with these conditions, and therefore, how they could or should be supported.

**WHAT THIS STUDY ADDS**

- This study provides a richer understanding of the lived experiences of people with INOCA. It highlights the significant impact on quality of life and challenges associated with everyday living and navigating the healthcare system. It illustrates an urgent need to improve awareness, treatment and support.

**HOW THIS STUDY MIGHT AFFECT RESEARCH, PRACTICE OR POLICY**

- The study provides a rationale for improvements in education and training for first-line staff including paramedics, accident and emergency and cardiology professionals to improve the experiences of people with INOCA who present with acute symptoms. It highlights the value of shared decision-making and multidisciplinary care for people living with INOCA and the importance of involving people with lived experience in the design and delivery of care pathways and support.

## Background

Coronary artery disease is traditionally characterised by obstructive atherosclerosis. However, up to two in five people experience symptoms of myocardial ischaemia without obstructive coronary artery disease.^1^ This condition can be caused by a range of underlying pathologies including coronary endothelial dysfunction, microvascular remodelling, microvascular and epicardial spasm and vasomotor abnormalities.^2^ A range of medical terms is used to describe distinct aspects of these conditions, including microvascular angina, coronary microvascular dysfunction, vasospastic angina, coronary vasospasms, coronary artery spasms, prinzmetal/variant angina and angina/ischaemia with no obstructive coronary arteries (ANOCA/INOCA). Women are disproportionately affected, particularly over the age of 40 years.^3 4^

While the prevalence of INOCA is increasingly described, the nature of this condition is not.^2^ To date, the primary focus has been to develop a diagnostic consensus.^5–7^ However, the relationships between symptoms, mechanisms, diagnosis and treatment responses are incompletely understood.^8^

Increasing investment in understanding diagnosis and treatment is a welcome development given the significant implications for the health and well-being of those affected. Self-reported survey data highlight a substantial adverse impact on quality of life including physical, social and mental health.^9 10^ However, there is a paucity of qualitative evidence about patients’ experiences of living with these conditions. This is important for understanding what is needed to improve support, long-term management of the condition and to optimise quality of life. This study investigated the lived experience of people with INOCA to gather qualitative evidence which will later inform the design and implementation of care pathways and surrounding support.

## Aims

We carried out a qualitative investigation to (1) explore the lived experiences and (2) understand the support and rehabilitation needs of people living with a confirmed or presumed diagnosis of INOCA. Our analysis generated two sets of themes which are presented in two separate manuscripts. This manuscript presents findings relating to the first aim, that is, peoples’ lived experiences of INOCA conditions.

## Methods

Interviews were conducted with English-speaking adults aged >18 years who self-identified as having INOCA. This was defined as microvascular angina, coronary microvascular dysfunction/disease, vasospastic angina, coronary vasospasms, coronary artery spasms, prinzmetal or variant angina, ANOCA/INOCA. Considering previous research detailing the challenges of diagnosis and advice from a patient with lived experience of INOCA, we did not ask participants to provide objective evidence of a definitive diagnosis but to self-identify based on their current medical advice. Information about the study was shared by patient representatives via online patient support groups, personal social media accounts and included in a British Heart Foundation ‘Heart Voices’ newsletter.

People expressing an interest in the study were provided with details about the research and invited to an informal telephone discussion with the interviewer (HH) to discuss the research aims and procedures. Those wishing to proceed completed a written consent form returned via email or post prior to interview. Recruitment ceased after interviewing 17 participants, when thematic saturation was reached.^11^

### Patient and public involvement

Study materials including the protocol, participant information sheet and consent forms and interview guides were developed under the guidance of a person with lived experience of INOCA. Discussions highlighted the importance of monitoring participants for signs of stress caused by the interview and ensuring that participation did not trigger chest pain. This prompted the inclusion of a specific distress protocol to guide the interviewer. Other improvements included the refinement of language to ensure that a range of terms used by patients to describe their condition were included.

### Interview procedures

An interview guide taking the form of a workbook (online supplemental material 1) was sent to participants prior to their interview. This provided participants with space to reflect and make notes on their lived experience prior to the interview, although it was stressed that doing so was entirely optional. The workbook included broad open questions about current health concerns and the impact of INOCA symptoms and/or diagnosis. It was followed by more specific questions about managing lifestyle, medications and psychological health. The workbook was subsequently used as a semistructured interview guide.

10.1136/openhrt-2023-002569.supp1 Supplementary data

Interviews were conducted via Zoom or Microsoft Teams, with three interviews carried out by telephone to suit participants’ preferences or connectivity requirements. All interviews were conducted by HH, an experienced female qualitative psychology researcher. Interviews were audiorecorded and limited to a maximum of 1 hour to limit cognitive or emotional burden for participants.

### Data analysis

Recordings were transcribed verbatim by a professional transcription service. Transcripts were sent to participants for review; one participant responded with minor clarifications which were included in our analysis.

Reflexive thematic analysis with inductive, semantic coding^12^ was used to analyse the data. Consistent with recommendations, we did not set out to achieve intercoder reliability.^13^ Instead, two researchers coded the transcripts separately to encourage reflexivity and ensure our analysis considered different possible interpretations. Two researchers (HH and DP, both with postgraduate psychology qualifications and experience in qualitative analysis) reviewed 50% of the transcripts each. DP and HH independently developed preliminary coding frameworks presenting initial themes, which they compared, merged and refined. Further discussion with a third researcher (AC) supported the sense-checking of candidate themes. Preliminary themes were also shared with all interview participants at this point, who were encouraged to feed back any questions or views. Four participants responded, advising that the themes presented gave a fair reflection of their experiences. Final themes are presented below along with illustrative participant quotes.

## Results

Seventeen participants expressed an interest to participate in the study and subsequently took part in an interview. A further four people requested information but did not subsequently participate, and six people were deemed ineligible due to a history of obstructive coronary artery disease. Table 1 shows the gender, age, self-reported diagnosis of participants.

**Table 1:** Participant characteristics

Characteristic	N
Participants recruited	17
Gender	
Male	2
Female	15
Age	
31	1
36	1
41	1
47	1
50	1
51	1
52	1
53	1
56	1
58	2
59	2
61	1
68	2
69	1
Self-reported diagnosis	
Coronary vasospastic angina (CVA)	1
Microvascular angina + suspected vasospastic angina (MVA, VSA)	1
Coronary artery spasm (CAS)	4
Microvascular angina (MVA)	5
Vasospastic angina (VSA)	1
Coronary microvascular dysfunction + coronary artery spasm (CMD, CAS)	1
Vasospastic angina / Coronary artery spasm (VSA/ CAS)	1
Microvascular angina + vasospasms (MVA)	1
Variant angina	1

Five themes were developed to reflect participants’ lived experience:

- Living with fear and uncertainty.
- Disruption to normal life.
- Psychological impact of the journey to diagnosis.
- Lack of clarity over treatment and management.
- Coping and supportive strategies.

Themes are presented alongside illustrative participant quotes.

## Theme 1: living with fear and uncertainty

Participants gave rich, detailed accounts of their experiences of living with a set of conditions that are highly individual but commonly underpinned with significant fear and uncertainty. It was clear from the interviews that there was no ‘typical’ INOCA presentation. Participants’ symptoms ranged in length, frequency, regularity, intensity or severity and predictability. They described either isolated, infrequent, regular or clustered ‘attacks’ involving chest pain, which was sometimes mild and relatively manageable, and at other times escalated until it was intolerable and required emergency medical intervention. For some participants, the chest pains were sometimes accompanied by breathlessness and sometimes the attacks were followed by fatigue and/or brain fog:

You can be really, really seriously bad for weeks and weeks and weeks at a time and then you think oh it’s eased off… you start to relax a little bit and then it’ll strike again. So, it can be anywhere between a couple of weeks to a couple of months‥‥ If I went [to hospital] every time I had pain I’d be there every day (P8; CMD/CAS)

…I’m not a person that has loads every day or week or whatever, it tends to come in clusters, and I can go months and months apart…. it can be very frightening and excruciating and they can range from mild to absolutely I’ve nearly passed out… We’ve had to have the ambulance out several times (P11; VSA/CAS)

There was a wide variation in predictability of these attacks. Some participants were clear about what triggered their chest pain; cold weather, physical exertion, emotional stress and/or food were variably cited as triggers, but this was individual, and no specific trigger was cited universally. Other participants felt that the attacks were largely unpredictable:

With this, you can just be sitting, not even thinking about it, and it can strike. It’s the not knowing. It’s the unpredictability is as disabling as the actual pain and fatigue (P8; CMD/CAS).

The unpredictability of these conditions, alongside the potential for attacks to become severe and highly distressing, left many participants living with substantial fear and uncertainty. The intensity of the chest pain was reported by one participant as equivalent to childbirth, and by several others as equivalent to a heart attack:

…it was… absolutely terrifying… I never in a million years thought it was going to be that severe the pain, and that sudden… that’s been the toughest thing to deal with, I think, and just that fear. (P3; CAS)

Many participants also highlighted concerns regarding their long-term health. This included questions about whether attacks were causing long-term damage to their heart, concerns about ageing and the onset of associated comorbidities:

…every time I have a spasm or a really bad spasm, especially at night because I can’t do anything about that, you know, it’s restrictive blood flow to your heart therefore is it damaging it?… Because I didn’t have an unhealthy heart and I don’t want to develop one. (P16, Variant angina)

## Theme 2: disruption to normal life

For most participants, the onset of their condition had a profound impact on their everyday lives, relationships, work and employment. Many reported needing to take long-term breaks from work or retiring altogether as a result of their symptoms, which had been difficult to accept and for some had challenged their sense of identity. Others had reduced working hours or were considering doing so. Participants expressed concerns about maintaining employment while managing their condition, particularly avoiding physical exertion or emotional stress and worried about becoming too ill to work in the future:

I do feel fearful about going back to work… I don’t know how I’m going to be back working and I do feel a bit, well quite fearful really of the stress and pressure. (P17; MVA)

Social relationships were affected in different ways. Some participants described their condition bringing them closer to their family through their need for support, although this was accompanied by regret about the impact on those close to them:

… it’s drawn us together as a family and we communicate as a family in a way we wouldn’t have done before. So that’s the positive bit… But there’s a lot of negative as well, living in pain is pretty tough. And my family have to watch me in pain. (P1; CVA)

Other participants described challenges in their relationships where family and friends did not understand their symptoms or their severity:

For me, it’s more emotional relationships, as I say there has been a real breakdown in mine…When I come back in from a school run if he’s about and I’m there laid on the kitchen floor clutching at my chest and I just think he thinks I’m being dramatic (P14; MVA/Vasospam)

Some participants reported finding it difficult to commit to, or fulfil social obligations and worried about the effect on their relationships:

…this weekend we’re off to [location] for a week, which is the first time we’ve been away for several years, but it makes it quite difficult… to plan the good stuff, because you’re always kind of like yeah but what if this happens? (P7; MVA)

## Theme 3: psychological impact of the journey to diagnosis

Participants’ descriptions of how they came to a diagnosis, whether presumed or confirmed, were often fraught with stories of being misdiagnosed, misbelieved or mistreated. Challenges included the invasive diagnostic tests that not all hospitals were willing or able to perform and the fact that many cardiology and other medical professionals were not aware of or accepting of these conditions:

So again back and forward, back to see a different cardiologist, oh she wasn’t having any of it, I have been a cardiologist for X amount of years, I know my job and this cannot be your heart, it’s psychosomatic again…. and this went on, gosh through my 20s, through my 30s, meanwhile I was in A&E goodness knows how many times with this pain. And they just dismissed it… They did a standard angiogram and said oh your main coronary arteries are normal…this is not your heart (P8; CMD/CAS)

So a lot of people will go into A&E. They’ll have baby rises in troponin, under the threshold…. it’s very difficult to catch a spasm in action (P1; CVA)

Participants described receiving mixed diagnoses depending on individual consultants. They also described a hierarchy of opinions at play, with many having to seek increasingly senior second opinions from specialists to establish their diagnosis. Their own opinion, as the person experiencing the pain, was often not prioritised:

And I went to see some really prominent cardiologists who misdiagnosed me… And that angiogram result, I felt absolutely validated because I thought I’m right. I am right and I was right about my body. (P1; CVA)

I did question my sanity at times and I actually thought so this is what it feels like to be mad. Because you have a sense of yourself and what you’re feeling, but all those around you perceive differently. And that had quite a huge impact on my mental health. (P2; MVA/VSA)

Several participants described distressing incidents of being admitted to hospital with extreme chest pain and yet not being taken seriously. Advocating for oneself among sceptical healthcare professionals, during or following an episode of intense pain was described by some participants using emotive language including ‘trauma’ and ‘gaslighting’:

And then when I’m being told that it’s not my heart that triggered off other things, you know, stress, anger, and then actually it’s like PTSD afterwards (P8; CMD/CAS)

The experiences reported by participants indicate an urgent need for improved awareness, understanding and training among both A&E and cardiology professionals:

And I won’t even go into A&E for whatever anymore because there’s no point… they don’t understand it and as far as they’re concerned my ECG is fine and my troponin is negative. (P15; MVA)

A few participants, especially those with longer histories of INOCA, acknowledged that there may be increasing awareness, or a shift in attitudes. This may be reflected in participants with more recent onset of symptoms attaining a quicker diagnosis:

… now the response I’m getting is actually you are a group of patients that we have neglected and it’s about time we looked after you a lot better… That was what the cardiologist said to me last time. So there is a growing awareness. (P1; CVA)

## Theme 4: lack of clarity over treatment and management

Participants expressed frustration, disappointment and concern at the lack of clarity over treatment and management pathways. Many described frustrating experiences of trial and error with medication, and a lack of confidence in the sufficiency of research evidence or guidance about managing their condition:

And because of the lack of research or the lack of appropriate treatment, you can try different drugs but there’s no cure and there’s no targeted treatment at the moment. So sometimes it’s a hit or a miss. (P2; MVA/VSA)

Participants whose condition was managed in primary care rather than cardiology tended to report that general practitioners (GPs) were open-minded and willing to help, but in most cases the GP lacked specialist knowledge of the condition and had no authority to request additional cardiology tests. Participants who reported more satisfaction were those who felt that different healthcare professionals were working in a coordinated way for them:

[My GP] is very helpful and we are working together. We worked together before I found a cardiologist which would listen to me… and now we’re continuously working with cardiologist and my GP… to tailor treatment (P13; CMD)

## Theme 5: coping and supportive strategies

Participants highlighted a range of factors that helped them manage and cope. There was a sense of empowerment for most in getting a diagnosis (sometimes even just presumed). Although many expressed a desire for more research to understand the underpinning causal mechanisms of the condition, they acknowledged that diagnosis provided an opportunity to educate themselves about what was happening to them:

Power! At least you know what you’re dealing with, you know what’s going on (P11; VSA/CAS)

Establishing protocols around treatment or medication was another way to regain some control. This was aided by supportive healthcare professionals, willing to work with the participant over time to understand their unique presentation of symptoms and triggers alongside their other comorbidities and circumstances:

… because with my cardiologist and GP, we were trying and looking what’s happening, the treatment, it’s not hundred percent yet, but it seems like I understand more these triggers and what to expect, how it affects me as a whole, and also how different medicines work. So I think I am… on the way to become more stable and more in control of my condition. (P13; CMD)

Common self-management strategies were another way to improve perceived control. Activities included low-impact physical activity such as swimming and walking, taking care of pets, gentle forms of exercise such as yoga or tai chi, meditation and mindfulness. Participants emphasised the importance of these activities for managing their emotions and stress levels, to reduce the likelihood of attacks and also to maintain positive well-being. Emotional well-being included accepting their condition and how it had changed their life (although reluctantly), realigning priorities, reducing their exposure to external stressors where possible and distinguishing between things within or beyond their control:

… some of the ideas that I’m just trying to implement myself… making sure I have a little walk each day or something, if I can do that, and prioritising self-care and things for me and sort of realising that actually it is OK to put myself or my family forward (P7; MVA)

Finally, participants cited the value of peer support. Many had been recruited from online, peer-led support groups and commented on how vital these were as a source of emotional support, advice and information about their condition. Some described peer support as a lifeline in helping them to secure or understand their diagnosis:

I’ve joined a group on Facebook who also have these spasms, so it’s kind of opened it up to a network of people and we can share stories and tips of how we can improve our way of life and things to look out for and just stuff like that. It has helped in that respect quite significantly. (P4; CAS)

Finding the support group has been such a relief to have an outlet to speak to people who understand where others don’t… And I’ve seen so many people say they’re so thankful for the group, like it’s literally saved so many people going through such tough times, because… it does change your life (P9; CAS)

## Discussion

### Acute experiences and psychological impact

Our findings provide important insights into the substantial impact of INOCA on quality of life, building on previous research^14 15^ by offering rich descriptions of the realities of living with these conditions. For most participants, the adverse psychological impact of their condition was a consequence of experiencing unpredictable physical ‘attacks’ combined with the distress associated with a lack of support and at times, challenging confrontations from healthcare professionals. There are some similarities with the experiences reported by people living with other medically unexplained symptoms (eg, fibromyalgia) who face a lack of clear advice and experience significant difficulties gaining a diagnosis.^16^ However, for people with INOCA the relapsing nature of the condition meant that many had on multiple occasions required emergency medical care. A dangerous consequence of these bad experiences in healthcare settings was the tendency for some people to resist seeking medical help in the future. Given the difficulties in differentiating ischaemic symptoms due to coronary spasm from those arising due to acute coronary thrombosis or other acute cardiac problem, this could leave people vulnerable to experiencing a definitive cardiac event.^15^ It also prevents a pattern of their attacks being recorded systematically over time, making it harder to establish or advocate for a diagnosis. A systematic review of cardiac disease-induced posttraumatic stress disorder (PTSD) suggests that risk of PTSD is higher for patients with increased hospitalisation, more invasive procedures and more negative illness representations.^17^ Although the review did not cover INOCA specifically, the likelihood of repeated hospital admissions for people with these conditions, lack of clarity about diagnosis and the invasive angiograms they may undergo indicates a need for better assessment of possible PTSD and steps to reduce likelihood of hospital-induced PTSD. Our study highlights an urgent need for education and training for first-line staff including paramedics, accident and emergency and cardiology professionals to ensure INOCA is considered as a possible cause of chest pain, there is clarity about how to investigate it and people presenting with suspected INOCA symptoms are treated respectfully and empathically.

### Improving diagnosis

Most participants placed high value on getting a diagnosis, although not all felt that it was necessary to have this confirmed via coronary angiography. For some, a presumed diagnosis had been sufficient to enable them to start researching and understanding their condition, better positioning them to self-advocate and self-manage. The routes to diagnosis described by participants highlighted a pervasive lack of consensus among clinical decision-makers, where years of experience in cardiology were not necessarily indicative of more awareness about INOCA. An ongoing challenge in raising awareness and making diagnosis quicker and easier will be that there is no typical presentation of symptoms. This means that hypothetico-deductive reasoning may be flawed in this context^18^ and highlights the importance of empathy and shared decision-making, which aims to value the patients’ opinion in the diagnosis.^19^ Peer-aided judgement and/or centres of excellence could help to connect patients with specialists for diagnosis, although this raises challenges associated with equity of access. Ongoing challenges associated with access to a definitive diagnosis also have implications for research. Participants in the current study were invited to self-identify based on their current medical advice but future research could gather information from participants on their medical therapy, results of any invasive and non-invasive diagnostic tests, and may also consider collecting data on current disease grade. This might contribute to a clearer picture of how symptoms present and affect quality of life across the spectrum of INOCA presentations.

### Long-term management

Good quality, trusting relationships with clinicians were highlighted in this study as key to feeling supported. Participants had varied experiences whereby some had access to cardiology teams and others were under the care of their GP. A multidisciplinary approach where healthcare professionals worked together with the patient was most satisfactory. This needed to happen over time so that participants developed an understanding of their individual triggers and patterns of symptoms, and tailored care plans could be developed accordingly. Clinical trials in North America have found that specialist multidisciplinary care leads to significant improvements in risk-factor management, quality of life and depression in addition to fewer emergency department visits and hospitalisations.^20^ Our findings suggest that more clarity is needed around eligibility for ongoing cardiology care and how this can be managed alongside input from primary care professionals. Collaboration could be improved by providing pain education and other supporting information to empower patients and their healthcare professionals to identify and manage chest pain appropriately.

Peer support from other individuals affected by INOCA was a fundamental element of many participants’ support networks. Most had highly valued interactions with peers and participated in online support groups, which they had sought out themselves. These communities were a rich source of knowledge about managing conditions, advocating for diagnosis and providing essential emotional support which was lacking in the formal healthcare system. This is consistent with previous research evidencing the value of such pooled collective knowledge.^21^ Online patient communities are a key source of information about how to manage INOCA and should be directly involved in the design and development of future treatment and support.

### Strengths and limitations

- To our knowledge, this article is the first to explore the lived experience of INOCA using semistructured interviews.
- The study design enabled in-depth inquiry into lived experiences.
- All aspects of the study were designed under the guidance of a patient–public involvement member with relevant lived experience.
- Participants were recruited via social media and online support groups, thus it was not possible to capture the views of people who were digitally excluded.
- Participants’ experiences were limited to the UK and not necessarily reflective of other international healthcare systems.

## Data Availability

No data are available. Complete transcripts are not available to protect participant anonymity.
