# Supplementary material for: Living with myocardial ischaemia and no obstructive coronary arteries: a qualitative study
Source: Open Heart. 2024 Feb 8;11(1):e002569. doi: 10.1136/openhrt-2023-002569 (PMC10860068; doi:10.1136/openhrt-2023-002569)
Supplement: Supplementary data [file openhrt-2023-002569supp001.pdf]

Designing cardiac rehabilitation programmes for people with NOCAD – patient workbook

This workbook is designed to explain what cardiac rehabilitation is and help you think about how relevant it might be for you. You can read through this workbook and make notes before your interview but please remember this is optional. **We will go through the workbook together during the interview.**

Living with non-obstructive coronary artery disease (NOCAD) including:

Microvascular angina
Coronary Microvascular dysfunction/ disease
Vasospastic angina
Coronary Vasospasms
Coronary Artery Spasms
Prinzmetal/Variant Angina
Angina/Ischaemia with no obstructive coronary arteries (ANOCA/INOCA)

If you have one of the conditions above (either presumed or confirmed), which fall under the umbrella term of NOCAD, this means the blood flow to your heart may be restricted and that you experience symptoms of angina, like chest pain.

We want to understand what it is like living with any of the conditions above, that fall under the umbrella term NOCAD.

What are your biggest health concerns (if you have any)? Use the space below to reflect or make notes if you wish.

Have you noticed any positive changes or experiences since being diagnosed with NOCAD?

Cardiac prevention and rehabilitation programmes (cardiac rehabilitation) for people with NOCAD

Cardiac rehabilitation is a programme designed to support recovery and rehabilitation after a cardiac event. The programme should be tailored to each individual patient but usually includes a mix of exercise and education to support your physical and emotional wellbeing. We would like to understand how suitable current programmes are for people with NOCAD and the best way to offer them so that people will want to take part. Please think about how relevant each part of the programme is for you, and how we can make sure it meets your needs.

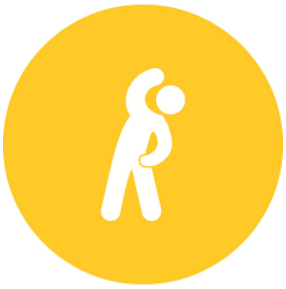

Exercise and physical activity

How do you currently manage this?

How confident are you about this (and why)?

How can we make this aspect of cardiac rehabilitation relevant and helpful for people with NOCAD?

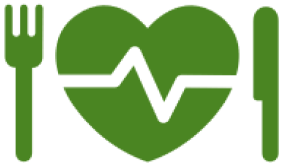

Diet and healthy eating

How do you currently manage this?

____

____

How confident are you about this (and why)?

____

____

How can we make this aspect of cardiac rehabilitation relevant and helpful for people with NOCAD?

____

____

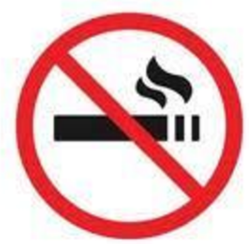

Stopping or reducing smoking

How do you currently manage this?

____

____

How confident are you about this (and why)?

____

____

How can we make this aspect of cardiac rehabilitation relevant and helpful for people with NOCAD?

____

____

Education and medical risk management

The education part of cardiac rehabilitation should be delivered using high quality teaching methods which suit different peoples' learning styles. It should use resources to help people to learn about their condition and how to manage it. Many topics could be covered. Please think about how relevant and/or important these topics are for you and make any suggestions about how we should deliver this part of the programme:

	**This is important (tick if yes)**	**Any notes or comments on the best way to support people with this topic:**
Managing NOCAD symptoms (e.g. chest pain)		
Managing risk factors such as blood pressure, lipids (fats in the blood) and glucose (blood sugar) on my own		
Using medicines to manage risk factors such as blood pressure, lipids (fats in the blood) and glucose (blood sugar)		
Using other non-medical approaches to manage risk factors such as blood pressure, lipids and glucose (e.g. physical activity, diet)		
Managing my alcohol use		
Managing smoking		
Managing my emotions and mental health		
Understanding when to seek help and where to go for that (from health professionals, and others in my social network)		
Managing daily activities such as housework, washing and dressing, gardening, and caring for my family		
Work and employment		

Resuming and maintaining sexual relations		
Surgery and implants		
Cardiopulmonary resuscitation		
Anything else?		

Emotional Health

People taking part in cardiac rehabilitation may experience many different emotional issues, and they should be offered screening and support for these. Please think about how relevant and/or important these issues are for you and make any suggestions about how we should deliver this part of the programme:

	**This is important (tick if yes)**	**Any notes or comments on the best way to deliver this part of the programme**
Anxiety and depression, post-traumatic stress (PTSD)		
Quality of Life		
Stress		
Social relationships		
Alcohol and substance use		

Understanding the best way to offer cardiac rehabilitation

When do you think people with NOCAD should be approached about cardiac rehabilitation?

Who and/or how should be people be approached?

When would you like / would have liked to start cardiac rehabilitation? (e.g. how soon after a cardiac event

---

If you have multiple hospital admissions, how should this considered or managed alongside when/how you are invited to take part in cardiac rehabilitation?

____

Cardiac rehabilitation is offered in different ways across the UK. Some people take part from home using the internet or working from a written guide. Other people attend a centre and complete the programme with a group of other patients, supervised by healthcare professionals. Which format do you think you would most prefer? Please put a tick next to the one you most prefer and cross next to the one you least prefer.

	Put a ✓ next to the one you most prefer and a **X** next to the one you least prefer	Note down any reasons for your choice and anything else you think we should consider about this
Home-based following a programme on the internet		
Home-based following a written guide or manual		
Centre-based (at the hospital or an NHS health centre)		
Centre-based (at a place in the community e.g. a leisure centre)		
Somewhere else?		

When would you like to attend cardiac rehabilitation?

	Put a ✓ next to the one you most prefer and a X next to the one you least prefer	Why would this time work best for you?
Morning		
Afternoon		
Evening		
Weekends		

Getting the maximum benefit from cardiac rehabilitation

We want to understand what people with NOCAD think they can gain from taking part in cardiac rehabilitation. Please complete the statements below:

I think people with NOCAD should take part in cardiac rehabilitation because:

____

____

If I took part in cardiac rehabilitation I would like to see these changes or improvements to my health:

____

____

Is there anything else you think we should consider when designing cardiac rehabilitation programmes for people with NOCAD?

____

____

Thank you for taking the time to take part in our study.
